# Gene bi-targeting by viral and human miRNAs

**DOI:** 10.1186/1471-2105-11-249

**Published:** 2010-05-13

**Authors:** Isana Veksler-Lublinsky, Yonat Shemer-Avni, Klara Kedem, Michal Ziv-Ukelson

**Affiliations:** 1 Department of Computer Science, Ben-Gurion University, Beer-Sheva 84105, Israel; 2 Virology and Developmental Genetics/Health Sciences, Ben-Gurion University, Beer-Sheva 84105, Israel

## Abstract

**Background:**

MicroRNAs (miRNAs) are an abundant class of small noncoding RNAs (20-24 nts) that can affect gene expression by post-transcriptional regulation of mRNAs. They play important roles in several biological processes (e.g., development and cell cycle regulation). Numerous bioinformatics methods have been developed to identify the function of miRNAs by predicting their target mRNAs. Some viral organisms also encode miRNAs, a fact that contributes to the complex interactions between viruses and their hosts. A need arises to understand the functional relationship between viral and host miRNAs and their effect on viral and host genes. Our approach to meet this challenge is to identify modules where viral and host miRNAs cooperatively regulate host gene expression.

**Results:**

We present a method to identify groups of viral and host miRNAs that cooperate in post-transcriptional gene regulation, and their target genes that are involved in similar biological processes. We call these groups (genes and miRNAs of human and viral origin) - *modules*. The modules are found in a new two-stage procedure, which we call *bi-targeting*, and is presented in this paper. The stages are (i) a new and efficient target prediction, and (ii) a new method for clustering objects of three different data types. In this work we integrate multiple information sources, including miRNA-target binding information, miRNA expression profiles, and GO annotations. Our hypotheses and the methods have been tested on human and Epstein Barr virus (EBV) miRNAs and human genes, for which we found 34 modules. We provide supporting evidence from biological and medical literature for two of our modules. Our code and data are available at http://www.cs.bgu.ac.il/~vaksler/BiTargeting.htm

**Conclusions:**

The presented algorithm, which makes use of diverse biological data, is demonstrated to be an efficient approach for finding bi-targeting modules of viral and human miRNAs. These modules can contribute to a better understanding of viral-host interactions and the role that miRNAs play in them.

## Background

MicroRNAs (miRNAs) are an abundant class of small noncoding RNAs (20-24 nts) that can affect gene expression by post-transcriptional regulation of mRNAs [1]. They typically base pair with sequences in the 3' UTR of mRNAs to inhibit mRNA translation or to promote their degradation. miRNAs have been shown to play important roles in various cellular and pathogenic processes, including development, cell death, immunological response, and carcinogenesis [2,3]. Since the functional characterization of miRNAs depends heavily on identification of their specific target mRNAs, numerous bioinformatics methods have been developed for this task (see e.g., [4-11], for a review see [12-14]).

**Figure 1:**
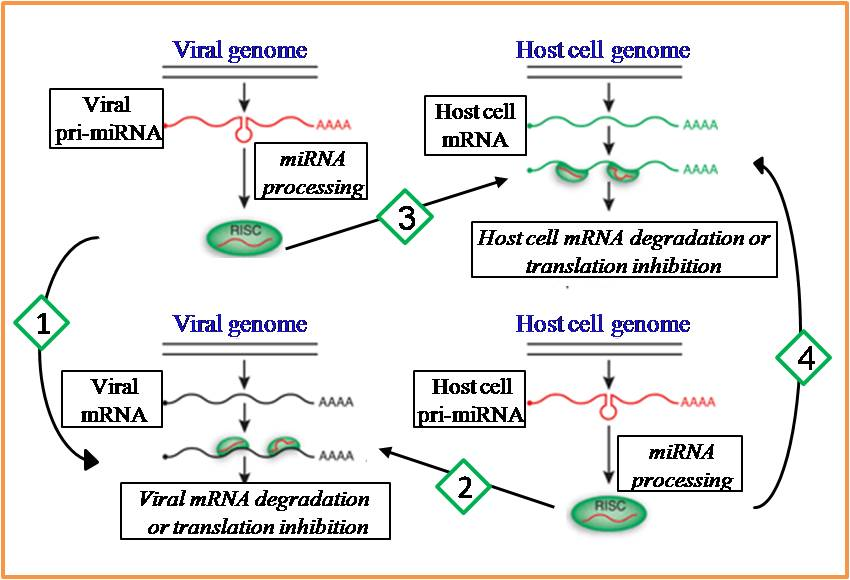
**Four scenarios for miRNA involvement in host-virus interactions**. (1) Viral miRNAs inhibit the expression of viral mRNAs. (2) Host miRNAs inhibit the expression of viral mRNAs. (3) Viral miRNAs inhibit the expression of host mRNAs. (4) Host miRNAs inhibit the expression of host mRNAs. In this case, viruses may regulate host miRNA expression and indirectly regulate host gene expression. (This figure is partially adapted from [81].)

In recent years, it has been shown that viruses also encode miRNAs [15,16]. Although for most of the viral miRNAs no functions have yet been described, it is conjectured that these miRNAs also take part in the complex interactions between viruses and their hosts, and several scenarios have been proposed [17] (see Figure 1, where scenario numbers are written in rhombi). In the *first scenario*, viral miRNAs regulate viral gene expression for maintaining, e.g., replication, latency, or evading the host-immune system. For example, miR-BART2 of EBV exhibits perfect complementarity to the 3'UTR of BALF5, which encodes the viral DNA polymerase [18], and such an interaction might be essential for maintaining EBV latency. In the *second scenario*, host miRNAs interact with viral RNAs, thereby inhibiting virus replication, e.g., miR-32 can limit the replication of the retrovirus primate foamy virus (PFV) in cell culture through an interaction with PFV mRNAs [19]. In the *third scenario*, viral miRNAs regulate host genes to induce a more favorable environment for the virus. The regulated genes are most likely related to antiviral response, apoptosis, interferon system, signal transduction, or cellular proliferation [20,21]. Stern-Ginossar et al. [22] showed that human cytomegalovirus (HCMV) miRNA, miR-UL112, inhibits the translation of a cellular gene MICB, which is normally activated when cells are subjected to severe stress, such as viral infection. MICB marks these cells for destruction by natural killer cells. The resulting absence of MICB protein protects HCMV-infected cells against lysis by natural killer cells.

Viruses may affect host miRNAs for their own advantage, by regulating up or down the expression of these miRNAs. This happens in the *fourth scenario *reported in [23]. In their research, host miR-17 and miR-20a were found to be downregulated following human immunodeficiency virus (HIV-1) infection. These miRNAs target the 3'UTR of host gene PCAF, which has been proposed to promote HIV-1 transcriptional elongation. Hence, this downregulation is needed for efficient replication of the virus.

In our work we will examine the combination of the last two scenarios, assuming that both human and viral miRNAs play an active role in regulating genes that either promote or inhibit viral replication or life cycle.

### Cooperativity of miRNAs in gene regulation

Previous studies show that one miRNA may have several target genes. Furthermore, one mRNA can be targeted by multiple miRNAs, reflecting *cooperative *translational control [5,24,25]. Experimental evidence indicates that multiple target sites (of one or more miRNAs) in the same 3'UTR can potentially increase the degree of translational repression [9]. In addition, microarray analyses [26] reveal that most of the miRNAs only modestly affect their mRNA targets. Recent findings show that some virus encoded miRNAs have sequence homology to their host human/mouse miRNAs, mainly in the seed region (see review [21]). This is one of many mechanisms that are used by viruses to exploit their host [27-30]. We were intrigued by an additional possibility in which viral and host encoded miRNAs target the same mRNA, possibly in different target sites, to promote gene silencing. It would make sense that viral miRNAs that share targets with human miRNAs may contribute to increasing the translational repression and tighten the regulation which already exists at a low level in the cell (by the host regulation machinery). This important question motivated us to study and predict the cooperativity of viral and host miRNAs on host genes.

**Figure 2:**
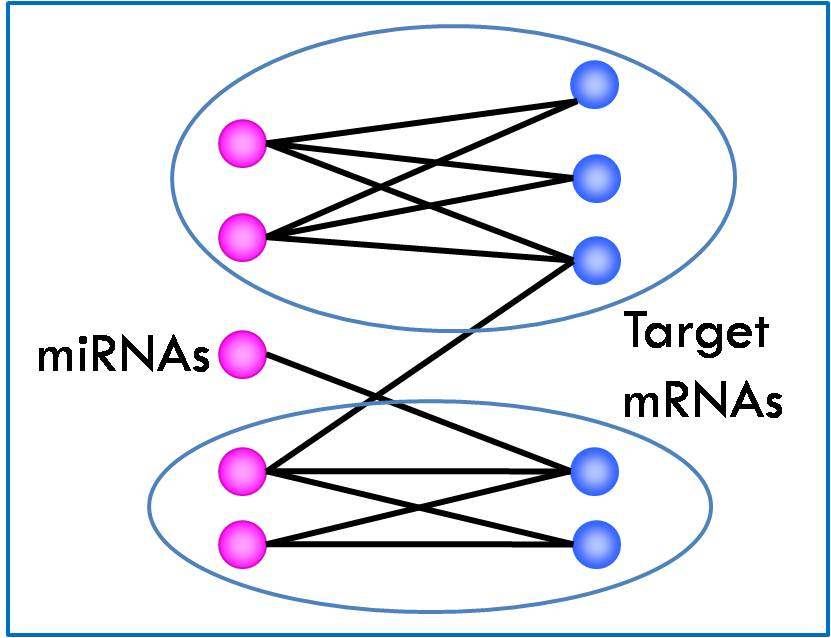
**Bipartite graph**. Bi-cliques in the graph are circled.

In this paper, we present a new computational method for examining cooperative effects of viral and host miRNAs on the regulation of host genes. We find modules consisting of both viral and human miRNAs, and their common target genes, that are involved in similar biological processes (according to GO). The mathematical formulation of our problem is an extension of a related problem, where the sought modules consist of miRNAs and genes of the same species. Below we present some studies that worked to solve the latter problem. Yoon and De Micheli [31], the first to address that problem, represent the multiple relations between miRNAs and target genes by a weighted bipartite graph, and find bi-cliques in the graph that represent the miRNA-mRNA modules (see Figure 2). They predict modules using target prediction based on sequence information.

Two other approaches combine several information sources to extract the modules, including predictions of miRNA target genes and their respective expression profiles. Joung et al. [32] apply a genetic algorithm, based on coevolutionary learning, to find an optimal miRNA-mRNA module. Tran et al. [33] use a rule-based learning method to identify the modules.

In general, finding bi-cliques in bi-partite graphs can be formulated as a bi-clustering problem (reviewed in [34]), which is known to be NP-complete. Therefore, most of the methods that address bi-clustering are based on heuristic approaches, which may miss good solutions. Alternatively, applying naive exhaustive enumeration to this problem is extremely time consuming to the point of being impractical. The problem becomes even more complicated when searching for cooperative human-viral miRNA modules. Directly applying one of the existing bi-clustering algorithms to a united set of both human and viral miRNAs may be insensitive to the contribution of viral miRNAs. This is due to the wide imbalance between the numbers of human miRNAs and viral miRNAs (hundreds of human miRNAs versus tens of viral miRNAs identified to date).

In order to be able to control the composition of the sought modules in terms of viral and human miRNA participants, a new type of clustering algorithm is needed. And indeed, in this paper we describe a bi-targeting algorithm which we developed (see Methods). We apply a branch and bound approach to develop and prune an enumeration tree of groups of human genes that are targeted by the same set of viral and human miRNAs. This algorithm is very efficient since we exploit the fact that the number of known viral miRNAs is relatively small, and that we demand a *quorum *on the number of miRNAs in the modules (a minimum number of viral and human miRNAs). This "pruning by quorum" allows us to apply enumeration in practical run time. We implemented our bi-targeting algorithm into a software system that combines a variety of biological data sources, such as: genomic sequences, miRNA expression measurements, GO annotations, and tissue context. The combination of several information sources in the task of module detection minimizes noise and errors accompanying each information source, resulting in more accurate results.

In addition, we developed a very flexible and efficient target prediction algorithm, which we briefly describe in the next section, and in more detail in the Appendix Section. We use it as a preprocessing step to the module search.

Using the bi-targeting system, we study the cooperative regulation of human and Epstein Barr Virus (EBV) miRNAs on human mRNAs in various lymphomas related to EBV. The significant modules are picked by a sampling procedure (described in the next section). We validate two of these modules by surveying information from the biological and biomedical literature. The rest of the significant modules can be found at http://www.cs.bgu.ac.il/~vaksler/BiTargeting.htm.

We believe that the results of this research may have far-reaching implications, as they may be applied to the diagnosis and treatment of viral infections, as well as for a wider understanding of viral-induced diseases and the role that miRNAs plays in them. Our study can contribute to the discovery of genes that regulate virus-host interactions, especially, but not only, in chronic infections, and can serve as targets for therapy [35,36].

## Methods

Our method consists of two stages: a target prediction stage and a module search stage. In the first stage, we perform all against all dynamic programming on two sets of sequences (miRNAs and mRNAs) in order to predict, for every miRNA in the set, its target mRNAs. In the second stage we look for modules that are enriched in some biological process, using the results from the first stage and information from Gene Ontology and the miRNA Atlas. We elaborate more on the second stage since this is the main goal of our paper. The interested reader may find a description of the first stage in the Appendix Section.

### Stage 1 - Predicting microRNA targets

The purpose of this stage is to perform an efficient genome-wide target prediction of all the miRNAs against all the possible subsequences of a set of mRNAs. miRNA target prediction is a well-studied problem, with numerous tools and approaches available. Each of these tools accommodates a variety of principles and features of target recognition, for example typical base pairing patterns, thermodynamic analysis of the miRNA-target duplexes, conservation of the target site among related species, and accessibility of the target site. For a review see [13,14].

The amount of the data we need to analyze is huge (thousands of genes and hundreds of miRNAs) and thus applying existing tools to this analysis would be too time consuming. Hence, we developed our own efficient tool for this task (which can serve as a plug-in filter to other target prediction tools). The method we propose extends the *threshold all-against-all *sequence alignment algorithm [37,38].

We store the miRNA sequences in a prefix tree (trie) and the mRNA sequences in a separate prefix tree (for details see the Appendix Section). We then apply dynamic programming to compute hybridization error between a prefix from the mRNA tree and a prefix from the miRNA tree, for each such pair. If, in one comparison, the number of errors we encounter is larger than a given threshold, then we stop comparing this pair and its descendants in the respective trees (*prune *the subtrees). Those miRNA-target pairs that survived the error threshold can be further checked for duplex energy by the *RNAduplex *program [39]. This results in linking each miRNA in our data set to its predicted target genes.

This method is efficient since it simultaneously checks sets of mRNA sequences (and, respectively, miRNA sequences) that share a prefix in the mRNA (respectively, miRNA) prefix trees. Moreover, it goes over all the duplexes, and prunes those with a hybridization error above a threshold, allowing a very efficient filtration.

### Comparison with existing approaches

We compare our target prediction method with three existing tools (those that provide downloadable code). The tools are miRanda [5], PITA [40] and RNAhybrid [10]. Our dataset consists of 873 experimentally validated miRNA-gene pairs out of 99 human miRNAs and 640 mRNAs (from miRecords [41]). We downloaded the 3'UTR sequences of these mRNAs from Ensembl Biomart [42], a total of 2183 different transcript sequences. In our results, an miRNA-mRNA pair is considered a hit if the miRNA is found to target one of the transcripts of the mRNA. We ran our target prediction method with the constraints listed in Table 1. miRanda was run with default parameters. The parameters of PITA and RNAhybrid were set to be close to ours (seed 2-8, and the maximal bulge size, in RNAhybrid was set to 6). We compare the results of these four methods in Table 2. The information which we compare includes: the total number of predicted pairs, the number of TP predicted pairs (sensitivity) and the running time. For our target prediction tool, the total number of predicted pairs falls within the ballpark of both PITA and miRanda. RNAhybrid, on the other hand, predicts twice as many pairs. As far as sensitivity goes, our results pretty much match that of PITA and outperforms miRanda by far. RNAhybrid gave the best sensitivity, (however this comes at the cost of a very high number of total predicted pairs). When comparing running times, our tool noticeably outperformed all the other engines, this in spite of keeping up competitive sensitivity and total number of predicted pairs. Recall that fast running time was the main objective for developing our fast filtration.

**Table 1:** Constraint parameters for the target prediction algorithm.

seed location	2-8
maximum GU pairs in seed	1

maximum GU pairs total	4

maximal number of mismatches/gaps	6

maximal size of bulge in the target	6

maximal size of bulge in miRNA	6

**Table 2:** A comparison between our method and other target prediction tools.

Tool	Total number of predicted pairs^*a*^	Number of TP predicted pairs^*b*^	Sensitivity	Running time
miRanda	22,857	309	35%	2 hours and 42 minutess

PITA	28,032	661	75%	5 hours and 10 minutess

RNA hybrid	43,693	731	83%	31 minutes

**Our method**	24,571	625	71%	**40 seconds**

^*a *^Out of 63,360 pairs. ^*b *^Out of 873 experimentally verified pairs.

### Stage 2 - Finding modules - the enumeration algorithm

In this section we describe our enumeration algorithm for finding modules that are statistically enriched in biological processes (GO categories). An important objective in our study is to ensure that the modules found by our method contain miRNAs that are mutually expressed and thus can cooperatively regulate gene expression. In order to ensure this condition, we use the miRNA Atlas [43]. This Atlas provides miRNA expression profiles in different conditions (i.e., different organ systems and cell types, of normal and malignant tissues). We focus on a set of conditions where both human and viral miRNAs (of the virus of interest) are expressed. Based on these data sources, we address the following problem.

*Given a GO category C, a miRNA atlas condition A, expression level threshold t, a p-value threshold p, the minimal number of human and viral miRNAs required in a sought module q*_1 _*and q*_2 _*(the quorums), respectively. Find all the modules composed of miRNAs whose expression in A is greater than t, which include at least q*_1 _*and q*_2 _*human and viral miRNAs, respectively, and whose intersection of the target-sets of human genes yields an enrichment p-value smaller than p in C*.

The enrichment is measured using a hypergeometric p-value [44]. The hypergeometric p-value measures the statistical significance of the overlap between the target genes and the genes in the considered category (for more details see the Appendix Section).

**Figure 3:**
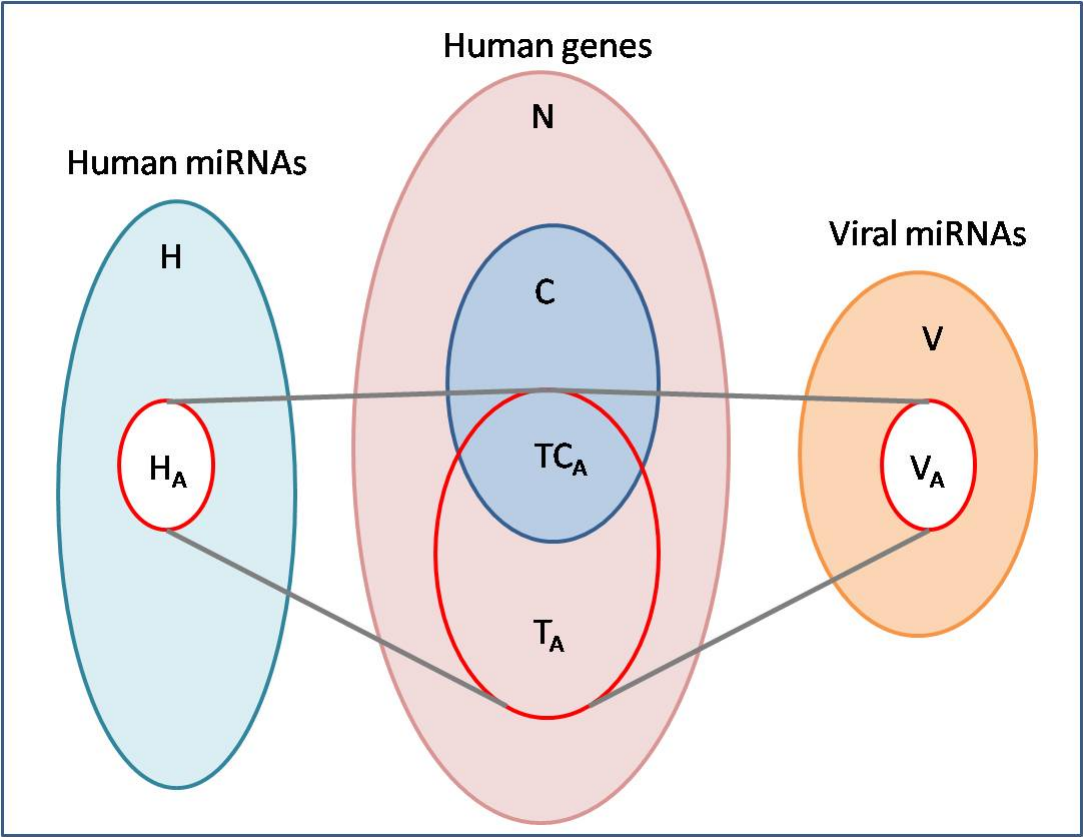
**An illustration of the connection between Atlas condition and GO category**. *H *is all the known human miRNAs and *V *is all known viral miRNAs. *H*_*A *_(*V*_*A*_) are the human (viral) miRNAs expressed in Atlas category *A*. They target gene set *T*_*A*_. *TC*_*A *_consists of the targets of *T*_*A *_that belong to GO category *C*.

For each GO category *C *and each Atlas condition *A *of interest, we perform the enumeration process as follows. Let *H *and *V *be the set of human and viral miRNAs. Let *H*_*A *_and *V*_*A *_be the subset of human and viral miRNAs expressed in *A*. Let *T*_*A *_be the set of their target genes (each gene in *T*_*A *_is targeted by at least *q*_1 _human and *q*_2 _viral miRNAs); and let *TC*_*A *_= *T*_*A *_∩ *C *(see Figure 3).

**Figure 4:**
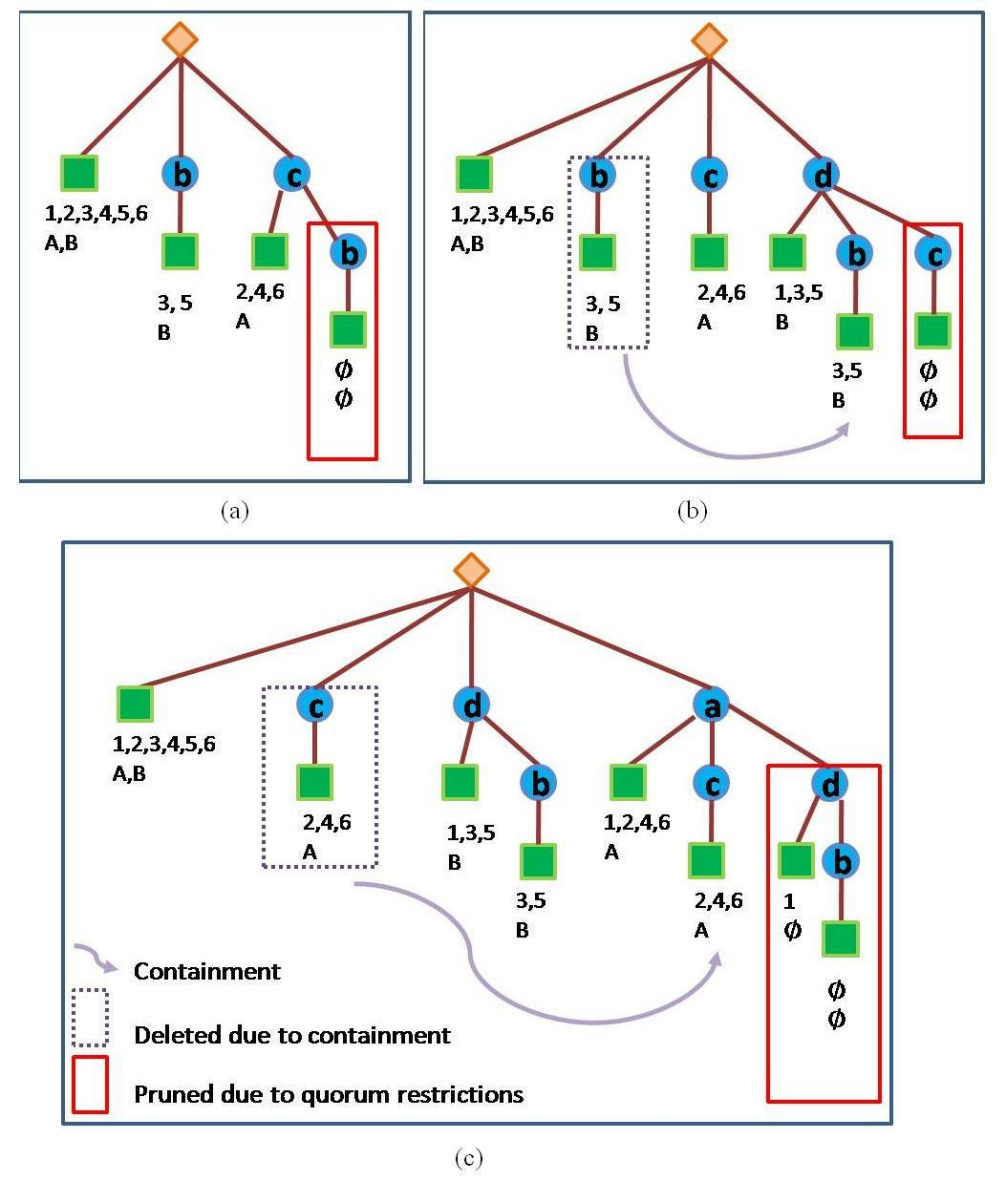
**The process of building the enumeration tree for target information in Table 3**. The rhombus represents the root of the tree. The circles represent the genes inserted into the tree. The squares represent the leaves, which store two sets of miRNAs - human and viral. These miRNAs are common "targeters" to all the genes in the path from the leaf to the root. Viral miRNAs are labeled with uppercase letters and the human miRNAs are labeled with numbers. The rectangles indicate modules that are pruned from the tree for one of two reasons: (1) modules that break the quorum are in solid rectangles (in this example the required quorum is 1 viral miRNA and 2 human miRNAs); (2) redundant modules (which are fully contained in another module) are in dotted rectangles. The arrow indicates the containment, starting from the redundant module and ending in the bigger one. The three sub-figures (a), (b), and (c) show the insertion of genes *b *and *c*, *d*, and *a *into the tree, respectively.

**Figure 5:**
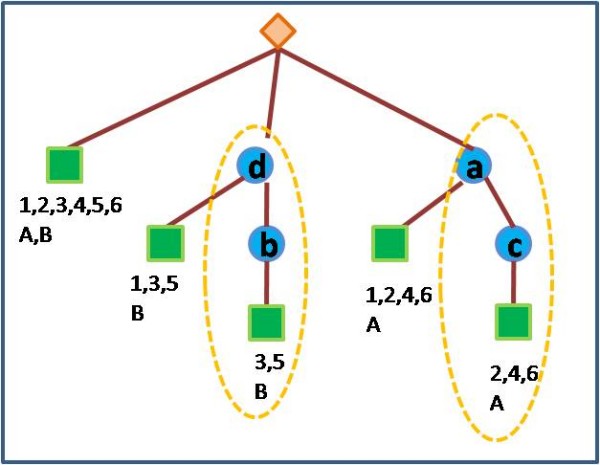
**The final enumeration tree**. The modules found by the enumeration algorithm (encircled by ellipses).

Our enumeration algorithm dynamically constructs and prunes an enumeration tree, which is built from the genes in *TC*_*A*_. A path in the tree represents a potential module. The module consists of the genes in the inner nodes of the path and a list of miRNAs (which target all these genes) in the leaf (see, e.g., Figures 4 and 5). At the end of the run of the algorithm, the enumeration tree stores in its leaves all the modules that satisfy the quorum constraints. The pseudocode of our algorithm is found in the Appendix Section. Below we supply an illustration of the algorithm applied on the data from Table 3, with the quorum constraints *q*_1 _= 2 and *q*_2 _= 1. We initialize the tree to consist of a root node and one dummy leaf node with all the human and viral miRNAs in *A *(see the leftmost leaf in Figure 4(a)). Genes are inserted to the tree one by one in order of increasing number of hits of human miRNAs on the gene (*b*, *c*, *d*, *a*). Each inserted gene, *g*, is connected by an edge to the root and becomes a root of newly generated copies of all its preceding siblings in the tree. The leaves of the new subtree contain intersections of *g*'s miRNA sets (human and viral) with the corresponding sets in the siblings' leaves. When these intersections do not satisfy the quorum constraints we prune the unsatisfying edge from the tree. (See the node for gene *b*, and then for gene *c*, in Figure 4(a). The leaves of the subtree rooted at *c *contain the intersections of the set of miRNAs hitting *c *with the set of the dummy leaf, and of *b*. Notice that the respective intersections of the miRNA sets hitting *b *and *c *are empty. Thus the edge from *c *to *b *is pruned from the tree).

**Table 3:** miRNA target information.

*Genes* *miRNAs*	*a*	*b*	*c*	*d*
human-miR-1	√			√

human-miR-2	√		√	

human-miR-3		√		√

human-miR-4	√		√	

human-miR-5		√		√

human-miR-6	√		√	

viral-miR-A	√		√	

viral-miR-B		√		√

total human miRNAs	1,2,4,6	3,5	2,4,6	1,3,5

total viral miRNAs	A	B	A	B

The columns correspond to genes (in *TC*_*A*_) and the rows to human and viral miRNAs (*H*_*A *_and *V*_*A*_, respectively). Cell (M, G) in the table is checked if miRNA *M *is predicted to target gene *G*. The last row provides, for every gene, its targeting miRNAs (numbers and letters corresponding to human and viral miRNAs, respectively).

In Figure 4(b) we show the tree after inserting *d *and making it the root of all its preceding siblings: the dummy node, *b *node, and *c *node. In the leaves of the subtree rooted at *d *are the intersections of the respective miRNA sets. Now two prunings occur in the enumeration tree. The original node *b *(in the dotted rectangle) and its targeting miRNAs, are now fully contained in the subtree rooted in *d*, thus *b *is now redundant and deleted from the enumeration tree. As in 4(a), the sets of miRNAs of *c *and of *d *are both empty and the edge from *d *to *c *is pruned (solid rectangle).

In Figure 4(c) we illustrate adding *a*, making it the root of the subtrees of its siblings. Intersecting *a*'s miRNA sets with the siblings' miRNA sets, resulting in deleting *c *(dotted rectangle) and pruning *d *(solid rectangle).

At the end of the process, the enumeration tree contains all the modules (see Figure 5). The leaves of the tree are traversed and their p-value is computed. The modules are then filtered (see the sampling procedure below) and reported according to ascending p-value.

#### The sampling procedure

To set a cutoff p-value for significant modules, we estimate the distribution of the hyper-geometric p-value scores for every <*A, C*> pair as follows. We sample, from the full set of miRNAs, two subsets of human and viral miRNAs, of same size as *H*_*A *_and *V*_*A*_, respectively. Then we find their target genes that satisfy the quorums, and intersect them with *C*. Next, we perform the enumeration algorithm on the genes in the intersection and store the hypergeometric p-values of the obtained modules. We repeat this sampling process 10,000 times, and compute the distribution of the obtained p-values. This distribution allows us to pick a significant p-value for our reported modules. We pick the p-value that is in the 0.01 quantile of the distribution to be the p-value cutoff for our reported modules.

## Results

We applied our system to the discovery of modules of genes targeted by human miRNAs and miRNAs of the Epstein Barr Virus. The Epstein-Barr virus (EBV) is a human herpes-virus that infects over 90% of the human population worldwide. EBV can infect B lymphocytes and epithelial cells, and typically establishes long-term asymptomatic latent infection in memory B lymphocytes. Nevertheless, EBV infection has oncogenic potential, which can result in a number of malignancies, including Burkitt's lymphoma, Hodgkin's lymphoma, nasopharyngeal carcinoma, and lymphoproliferative diseases, especially among immunocompromised individuals [45]. During the latent infection of EBV, only a limited subset of its genes is transcribed, allowing it to evade immune recognition.

Latency gene expression is classified into three groups: type I - expressing only nuclear protein EBNA1; type II - expression of EBNA1 and membrane proteins LMP-1, 2A, 2B; type III - six nuclear proteins EBNA-1, 2, 3A, 3B, 3C, LP, and three membrane proteins LMP-1, 2A, 2B. The noncoding RNAs EBER1 and EBER2 and a set of EBV-encoded miRNAs are differentially expressed in all three forms of latency [46].

### Datasets

The set of human and viral mature miRNA sequences was downloaded from the miRNA registry [47] containing 866 human miRNAs and 39 EBV miRNAs (mature sequences). The set of human 3'UTR sequences was extracted from the Ensembl's Biomart [42], database Ensembl 53. Each 3'UTR in this set is annotated with an Ensembl gene and transcript ID.

The gene ontology (GO) associations and ontology structure files were downloaded from [48]. Each 3'UTR was attributed to the corresponding gene name in the ontology file, using the Ensembl gene and transcipt IDs.

The set of 3'UTRs was filtered to contain 3' UTRs from genes that are expressed in B cells (data taken from Ensembl Biomart) and that have at least one biological process annotation. This resulted in 7208 sequences (different transcripts) from 4773 genes. UTRs longer than 3000 nts, were shortened to 3000 nts. miRNA expressions were taken from the miRNA Atlas. Eight tissue samples from the Atlas, expressing both human and EBV miRNAs, were found to be relevant. Seventy-two GO categories (that contain 10-300 genes) related to apoptosis, immune response, signal transduction, proliferation, and cell cycle were chosen.

### The target prediction stage

In the first stage we ran all-against-all target prediction between the miRNA and 3'UTR sets described above. The target prediction was carried out with the constraints listed in Table 1, which are derived from the literature (e.g. [12,22,49]). In addition we checked duplex energy at the leaves of the prefix tree (see Methods). The duplex energy was normalized by the energy of the duplex formed between the miRNA and its complement, and if the resulting ratio exceeded a threshold of 0.4 the target prediction was accepted as positive.

**Figure 6:**
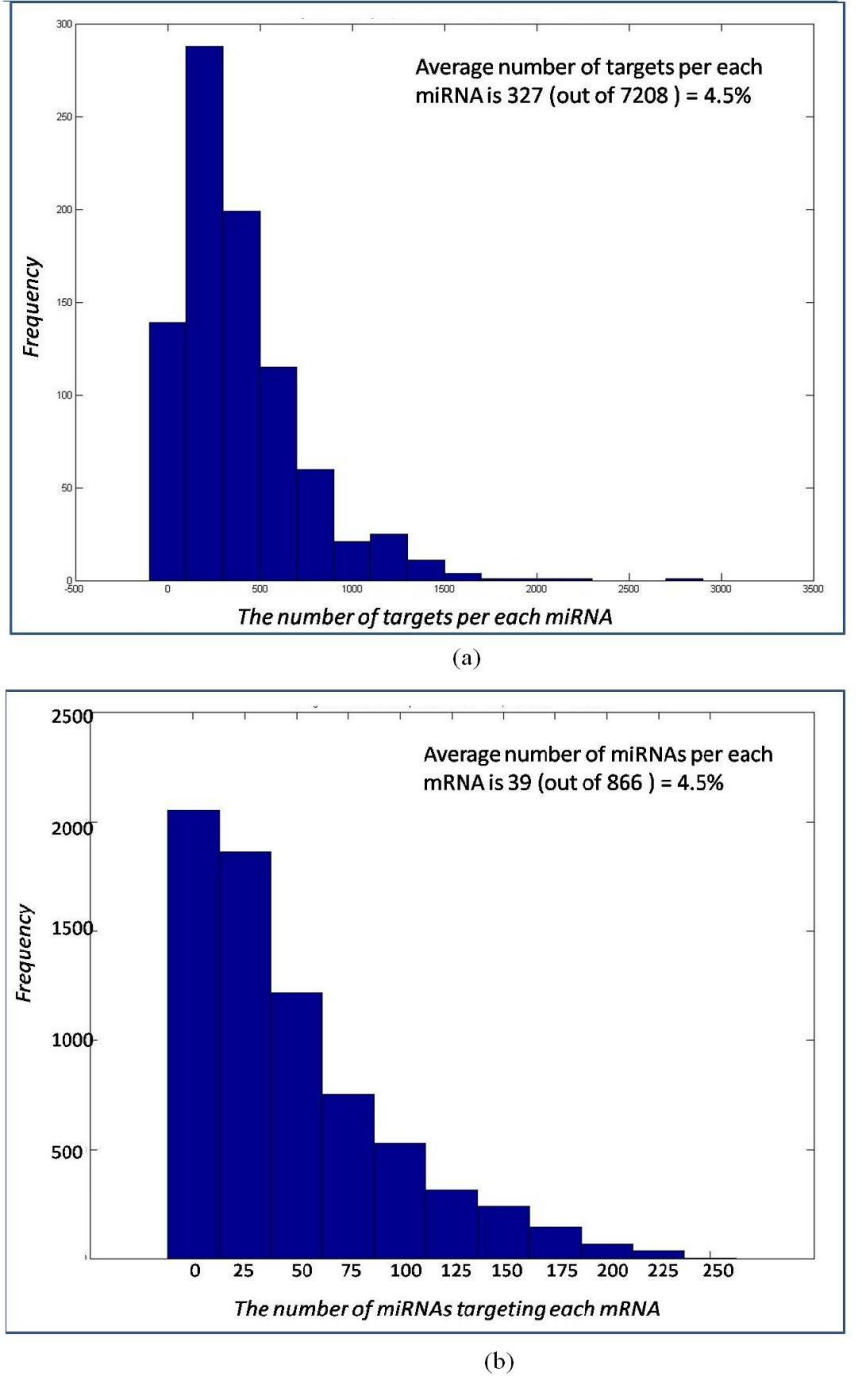
**Characteristics of the *human miRNAs/human mRNAs *relationships**. (a) Distribution of the number of target mRNAs per each miRNA. (b) Distribution of the number of miRNAs targeting each mRNA.

Running the target prediction on 866 human miRNAs (39 EBV miRNAs) and 7208 human mRNAs (3' UTRs) resulted in *283362 *human miRNA/human mRNAs (*11663 *EBV miRNA/human mRNAs) pairs. We analyzed some characteristics of the putative target-binding association (for the human miRNA-mRNA pairs). Figure 6(a) shows the distribution of the number of target mRNAs per each human miRNA, and Figure 6(b) shows distribution of the number of human miRNAs targeting each mRNA. According to this analysis, one human miRNA binds putatively to the 3'UTR of 327 mRNAs on average (4.5% of the total number of mRNAs in the dataset). Alternatively, one mRNA is targeted by 39 human miRNAs on average (4.5% of the total number of human miRNAs in the dataset). An analysis of the efficiency of our filter can be found in the Appendix Section.

### The predicted modules and supporting evidence

In this stage we used the target prediction results from the previous stage. The enumeration process was carried out on 72 GO categories versus 8 Atlas conditions, resulting in 576 <*A, C*> pairs, where *A *denotes an Atlas condition and *C *denotes a GO category. We found 54 modules that fulfilled the following quorum constraints, *q*_1 _= 2, *q*_2 _= 1, and had a p-value lower than the cutoff (obtained by a sampling procedure for each such pair. See Methods for details). Modules with fewer than two genes were filtered out. The expression level threshold *t *was calculated for each miRNA as the average expression among all the Atlas conditions related to *B cells*. Among the GO categories tested in our experiment, there are dependent categories that contain the same genes. Among Atlas conditions sometimes groups of expressed miRNAs overlap. In order to reduce the redundancy of the reported modules we combined identical modules from different GO categories and Atlas conditions. This procedure resulted in 34 modules. Below we present and analyze two selected modules that were found by our method. The full set of potential modules can be obtained at http://www.cs.bgu.ac.il/~vaksler/BiTargeting.htm.

**Figure 7:**
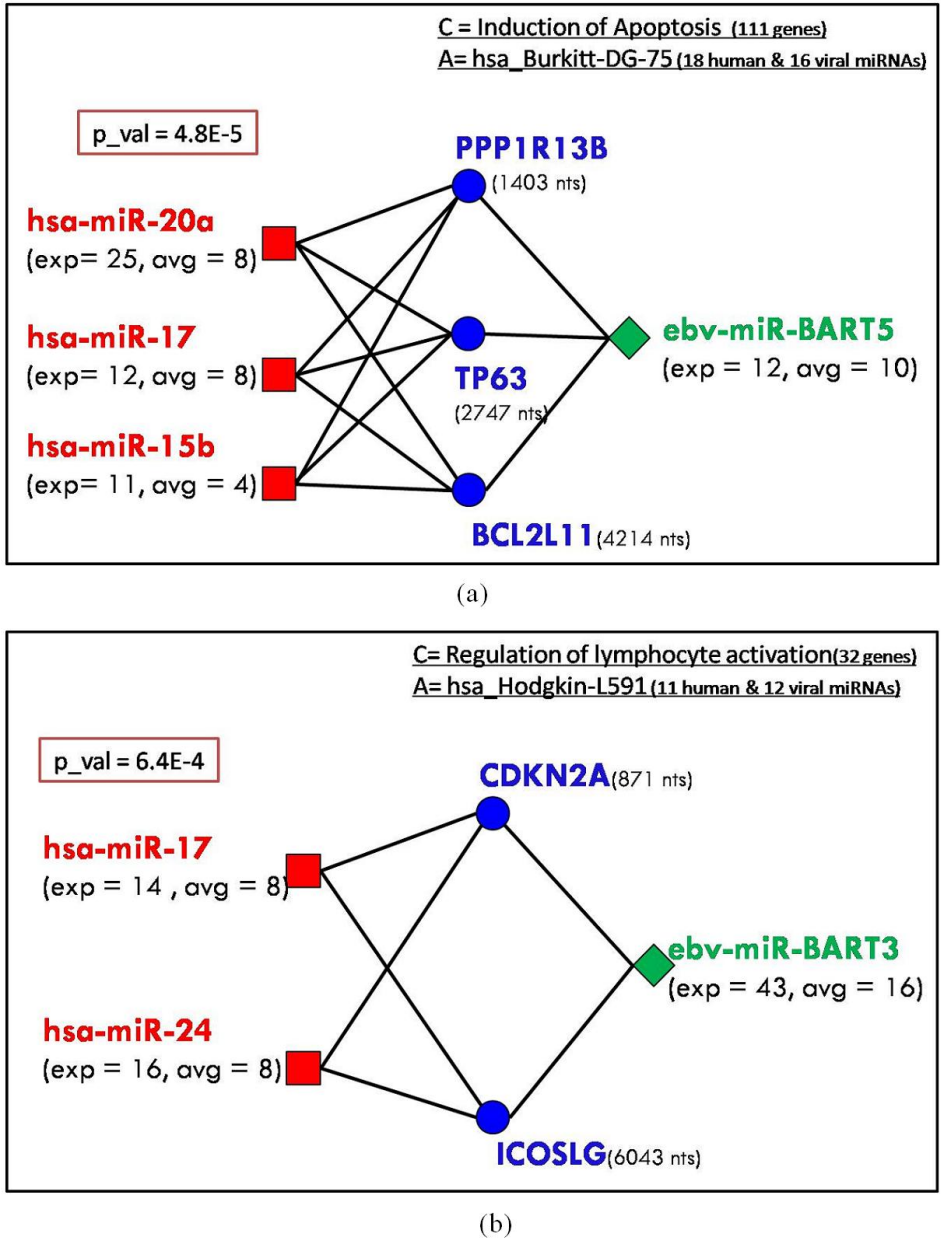
**Two predicted modules**. The length of the UTR is indicated in the parentheses near the gene name. The expression in the Atlas condition and the average expression among B cell conditions is indicated in the parentheses under the miRNA name. The Atlas conditions: (a) has_Burkitt-DG-75: Burkitt lymphoma cell line established from a 10-year-old boy, EBV+; (b) hsa_Hodgkin-L591: Hodgkin Lymphoma cell line, EBV+.

**Figure 8:**
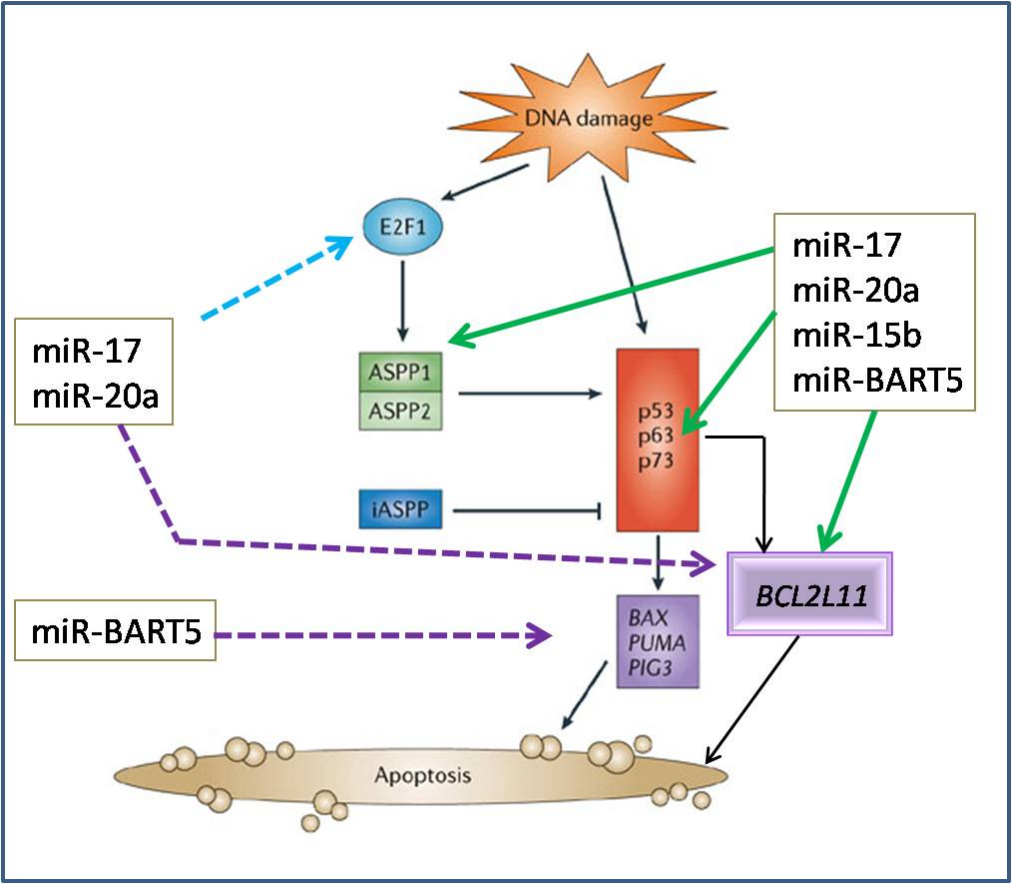
**The function of Module I genes in cellular apoptotic pathways, and the cooperative role of human and viral miRNAs in this cascade**. The dashed arrows indicate validated targets of the miRNAs, the solid arrows indicate the new regulation pattern discovered in our module (the figure is partially adopted from [56]).

***Module I***( see Figure 7(a)) was obtained from genes related to "induction of apoptosis" and miRNAs that are expressed in Burkitt lymphoma. The module consists of three human miRNAs, miR-17, miR-20a, and miR-15b, one viral miRNA, miR-BART5, and three human genes. A thorough literature search supported our predictions as follows. miR-17 and miR-20a belong to the genomic cluster miR-17-92. The cluster consists of six miRNAs that are tightly grouped within an 800 base-pair region of human chromosome 13 [50]. The miR-17-92 cluster first attracted attention following a series of observations linking these miRNAs to cancer pathogenesis, as it undergoes amplification in several types of lymphoma and solid tumors. It was also shown that these miRNAs are tightly linked to the functions of the E2F family of transcription factors, which are critical regulators of the cell cycle and apoptosis. Furthermore, O'Donnell *et al*. [51] validated that E2F1 is a target of miR-17 and miR-20a. Therefore, these miRNAs lead to decreased E2F1 protein and thus attenuate E2F induced apoptosis (see Figure 8).

The third human miRNA is miR-15b. This miRNA was recently shown to regulate cell cycle progression in glioma cells by targeting cell cycle-related genes, such as CCNE1 [52]. In addition, Cimmino et al. [53] reported that its analog miR-15a (along with miR-16-1) negatively regulates BCL2, which is an anti-apoptotic gene that is often overexpressed in many types of human cancers. The participation of this miRNA in this module suggests that this miRNA may also have oncogenic roles in this particular type of malignancy, by targeting genes that promote apoptosis.

The viral miRNA miR-BART5 was shown to target a human protein PUMA [54]. PUMA plays a role in apoptosis as a direct downstream target of p53, in addition to being able to initiate apoptosis via a p53-independent mechanism [55]. Down-regulation of PUMA by miR-BART5 may protect EBV-infected cells from virus-induced apoptosis. Furthermore, searching for targets of BART5 on the viral genome revealed 8 hits on the EBNA-LP protein. EBNA-LP promotes the expression of viral genes that expose the virus to the immune system.

Three human genes that were found to participate in this module are strongly related to apoptosis. PPP1R13B (also known as ASPP1) has an important function in cellular apoptotic pathways [56,57]. ASPP1 and ASPP2 (another member of ASPP family) are induced by the E2F1 transcription factor and cooperate with the tumor suppressor p53 and its family members p63 and p73 in trans-activating pro-apoptotic genes such as the BCL2-family member BAX, the BCL2-binding component PUMA, and p53-induced gene 3 (PIG3) (see Figure 8). Inhibition of endogenous ASPP function suppresses the apoptotic function of endogenous p53 in response to apoptotic stimuli [57].

The second gene in the module is BCL2L11(also known as BIM), which is a pro-apoptotic gene that regulates cell death in mature B cells. It is thought to initiate apoptosis by binding to and inactivating pro-survival BCL2-family members, such as BCL2 [58]. Deletions or methylation of the BIM locus are found in various human B lymphomas [59,60]. Furthermore, it was shown previously that latent infection of Burkitt Lymphoma cells with EBV results in a significant reduction in the expression of BIM [61]. A number of recent works ([62-64]) have also identified BIM as a direct target of multiple members of the miR-17-92 cluster (including mir-17 and miR-20a).

The third gene, TP63 (also known as p63), an important epithelial developmental gene, is a member of the p53 tumor-suppressor gene family. The p63 gene has two promoters, resulting in two different types of proteins with opposing functions, a p53-like protein containing the TA domain (TAp63) and inhibitory proteins lacking TA, called DNp63. TAp63 upregulates expression of proapoptotic Bcl-2 family members such as Bax and BCL2L11 [65]. p63 is capable of binding a series of p53-responsive promoters and can transactivate many p53 target genes. p63 activity is regulated by proteins such as ASPP1 and ASPP2 (see Figure 8), which also modulate p53 activity. p63 can mediate apoptosis in a manner similar to that of p53, and it was proposed that p63 is essential for p53-mediated apoptosis induced by DNA damage [66]. The cooperative regulation of human and viral miRNAs of the apoptotic pathway is demonstrated in Figure 8. The dashed arrows indicate validated targets of miR-17, mir-20a, and miR-BART5, and the solid arrows indicate the new regulation pattern discovered in our module. Our predictions may imply that miRNAs in this module target genes along the cascade that leads to apoptosis. In particular, these results predict that a primary function of miR-BART-5 may be to prevent p53 mediated apoptosis by targeting different mRNA transcripts.

***Module II*** (see Figure 7(b)) consists of miRNAs from the Hodgkin Lymphoma cell line, hsa-miR-17, hsa-miR-24, and ebv-miR-BART3, and genes from the "regulation of lymphocyte activation" category - CDKN2A and ICOSLG.

CDKN2A (also known as p16-INK4A) is a tumor suppressor that binds to the complex of cyclin D1 and cyclin-dependent kinase 4 to repress its ability to phosphorylate the retinoblastoma protein, and consequently, blocks cell cycle progression from G1 to S [67,68]. Inactivation of this gene has been shown in a wide variety of human cancers as a consequence of mutation, homozygous mutation, or promoter methylation [69-71]. In addition, it was shown that EBV oncoprotein LMP1 blocks the expression of CDKN2A, by promoting the CRM1-dependent nuclear export of Ets2, which is an important transcription factor for CDKN2A, thereby reducing the level of its expression [72]. Furthermore, human miRNA miR-24, which presents in this module, has been demonstrated to promote cell growth through repression of this gene [73]. miR-17, in addition to its function in attenuating E2F induced apoptosis (see module I), was shown to target CDKN1A (also known as p21), a gene that functions as a regulator of cell cycle progression at G1 [74].

The second gene found in module II is ICOSLG. This gene is expressed on monocytes, dendritic cells, and B cells and can be induced by inflammatory stimuli in peripheral tissue. Binding to ICOSL delivers a co-stimulatory signal for T cell proliferation and cytokine secretion [75,76]. Furthermore, this gene was shown to be important in several immune responses against pathogenic microorganisms such as bacteria, parasites and viruses [77-79].

The two modules presented have a tight connection to the function of the EBV, which establishes a long-term latent infection in the host cells. Thus, it is reasonable to assume that EBV will use its and the host's machinery, including miRNAs, for downregulating genes that lead to apoptosis or immune response. These modules supply evidence of the cooperation between human and EBV miRNAs in the task of preventing apoptosis, promoting cell growth, and evading the immune response. It is important to note that the target sites of the human and viral miRNAs on the UTRs of the genes in this module are different. Thus this cooperation, with multiple target sites, can lead to an increased degree of translational repression.

## Discussion and Conclusions

miRNAs represent a class of molecules produced by both viruses and their hosts that can benefit either the virus or the host, depending on the particular interaction. Viral miRNAs were discovered only recently, and functional relationships between viruses and viral or host miRNAs are only now beginning to be elucidated. A comprehensive understanding of the entire landscape of the miRNA-mediated host-virus interactions may uncover novel pathways that promote or limit virus replication. In turn, this knowledge may lead towards the development of effective antiviral therapy and could help guide drug design.

In this work we focused on the contribution of viral and host miRNAs in regulating host genes, and thus in promoting or inhibiting viral replication or life cycle. Our method searches for modules of miRNAs (host and viral) and their common host target genes that are involved in similar biological processes.

Our method is related to bi-clustering methods that have been used in various biological issues [34]. The bi-clustering approach groups rows and columns simultaneously in a two-dimensional data matrix. Alternatively, a data matrix can be viewed as a bipartite graph. Previous works, dealing with module search, represent one set of nodes as miRNAs and the second set as target mRNAs; the edges represent target relations. Since in our problem, the miRNAs are of two different sources (viral and human), we cannot use the existing methods of bi-clustering. Instead, our graph can be viewed as a two-sided bi-partite graph, and the goal is to find two sided bi-cliques.

In order to achieve this goal, we developed a new bi-targeting algorithm. The algorithm constructs an enumeration tree of human genes that are targeted by the same sets of miRNAs, and contains the modules at the end of its construction. Due to the application of a branch and bound technique, the algorithm is very efficient and enables, within a practical time, enumeration of all possible modules.

Our method combines a variety of biological data sources. Genomic sequences were used for the target prediction task. Tissue context information was used to narrow the list of human genes to genes that are expressed in the cells infected by the virus of interest (e.g. B cells in the case of EBV). miRNA expression profiles (from the miRNA atlas) and GO annotations enabled us to find potential modules consisting of miRNAs co-expressed in an Atlas condition (and have a potential to regulate cooperatively a group of genes) and genes with similar biological function from a GO category. The combination of several information sources in the task of module detection minimizes noise and errors accompanying each information source, resulting in more reliable results.

In addition, we developed our own method for target prediction, which extends the *threshold all-against-all *algorithm. The method performs an efficient genome-wide target prediction of all the miRNAs against all the possible subsequences of a set of mRNAs. Our algorithm was able to prune a large portion of the aligned trees by utilizing the fact that the number of errors in the miRNA-mRNA duplex is bounded by some threshold.

Note that the two methods we present in this paper are independent. One can use our target prediction algorithm as an efficient stand-alone target prediction system. In addition, our module finding method can use any source of target information, produced by any of the existing target prediction tools, using different principles of target recognition. The method can also be applied to other viruses, as long as their miRNA expression profiles are available (along with human miRNAs).

We applied our method to the discovery of modules consisting of human and viral (EBV) miRNAs and human genes. The identified modules display meaningful discoveries supported by the literature. Two of the modules are analyzed in detail in this paper.

Since not much is known about the function of viral miRNAs, finding modules that link the viral miRNAs and the human miRNAs, might help in understanding the role of viral miRNAs in viral infections. Thus the method developed in this work can be of help to better understand viral-induced diseases and the role that miRNAs plays in them.

## Authors' contributions

YSA presented the problem and constraint parameters. IVL, KK, and MZU developed the algorithms. IVL contributed to code development and data analysis, and found supporting evidence in the literature. All authors drafted the manuscript, and read and approved the final manuscript.

## Appendix

### Predicting miRNA targets: a filter based on error bounded complementarity

We perform a genome-wide target prediction of all the miRNAs against all the possible subsequences of a set of mRNAs in an efficient way. The method we propose extends the *threshold all-against-all *alignment algorithm [37,38]. All-against-all alignment problems can be solved using dynamic programming and suffix trees [37]. Sagot used a variant of this problem to extract approximate repeated motifs from a sequence or common motifs from a set of sequences [38]. In this work we extend this idea to the task of target prediction by using prefix trees (tries).

The trie data structure [80], *τ*, allows representation of the miRNA/mRNA sequences in an efficient way. The tree *τ *has the following properties: each edge of *τ *represents a character *c *∈ {*A*, *C*, *G*, *T/U*}, the characters represented by sibling edges are distinct, and the concatenation of the labels of the edges on a path from the root to a leaf represents the full string inserted in the tree.

For our target prediction system we construct two tries, one for the miRNA sequences and one for sequence segments of the (reversed) mRNAs. Next, we apply the branch and bound algorithm to find complementarities between sequences from the miRNA trie and the mRNA tries. In more detail, construction of the tries is as follows.

**Figure 9:**
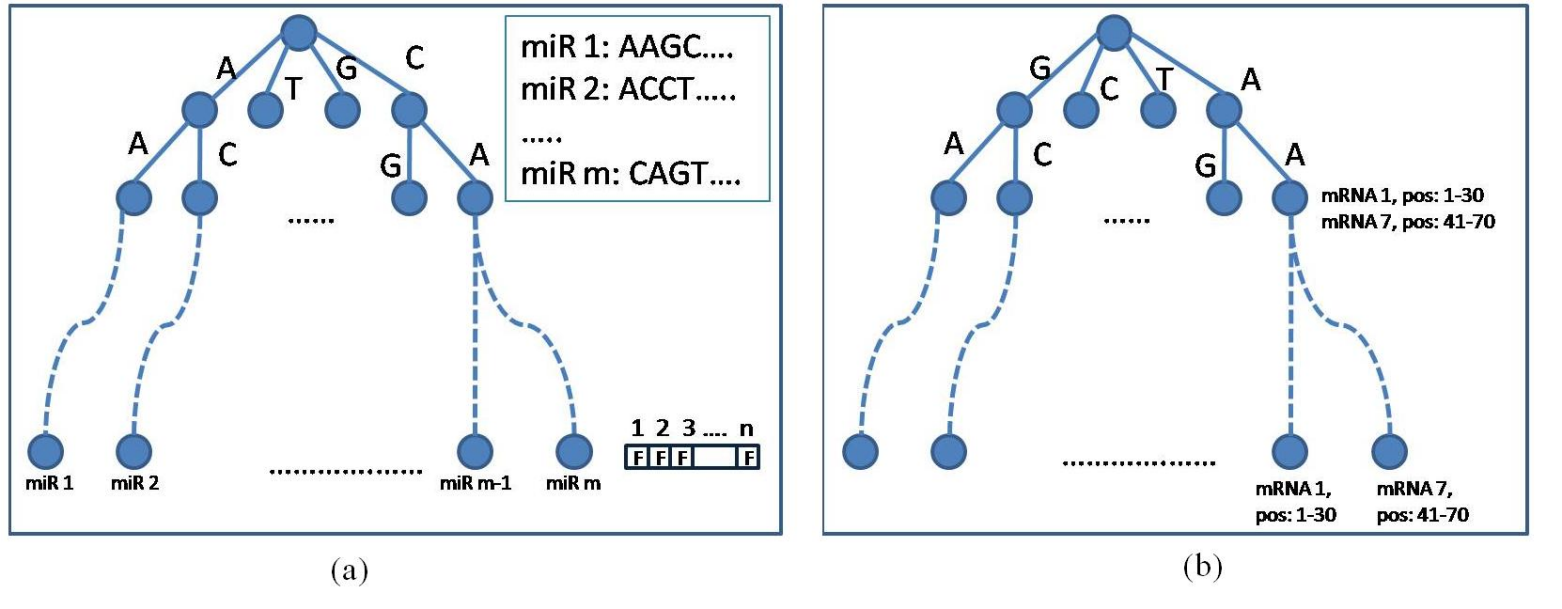
**The prefix tries**. (a) *τ*_*M *_and (b) *τ*_*G*_, miRNA and mRNA tries, respectively.

Let *M *be a set of *m *miRNAs, and let *G *denote a set of *n *mRNA sequences in the database for the organism under consideration. Consider the trees in Figure 9, *τ*_*M *_and *τ*_*G*_, which represent the miRNA and mRNA sequences, respectively. *τ*_*M *_is built from the miRNA sequences in 5*' *→ 3*' *orientation. A node *u *in *τ*_*M *_keeps the character of the incoming edge (the empty character in the root). A leaf node, which corresponds to an miRNA, keeps in addition a boolean vector *V *(*u*) initialized with *F *(for false). Each *V *(*u*) has *n *cells, one for each mRNA, and each mRNA has a running number. Cell *i *in *V *(*u*) indicates whether the *i*'th mRNA is the miRNA's predicted target (*V *(*u*) [*i*] = *T*).

*τ*_*G *_is built from mRNA subsequences, which are produced by partitioning the mRNA reversed (3*' *→ 5*'*) sequences into given size consecutive overlapping windows with a slide of 1-nt. The partitioning of the mRNA into windows ensures that every nucleotide in the mRNA will be examined as a potential starting point of a target site to one or more miRNAs. Since the mRNA sequence may have bulges when hybridized to an miRNA, we set the size of the window to be longer than the typical miRNA length, namely to 30 nucleotides. Each node in *τ*_*G *_keeps the same data as *τ*_*M *_. It also has a list of all mRNAs for which the path from the root of *τ*_*G *_to this node spells a substring of this mRNA (see Figure 9(b)).

**Figure 10:**
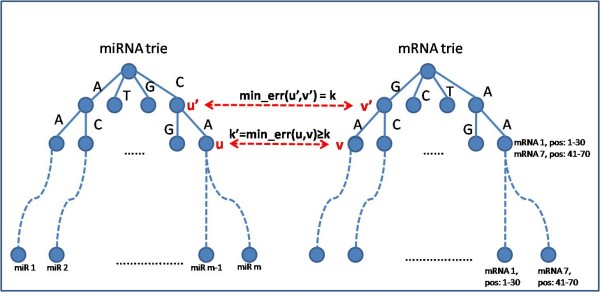
***min_err *monotonicity property**. The score *min_err*(*u*, *v*) is monotone with respect to the increase in the prefix length of the miRNA and mRNA sequences.

Our goal now is to measure the complementarity between all the miRNAs in *τ*_*M *_and the mRNA subsequences in *τ*_*G*_. In order to get an admissible branch-and-bound search, we use a target prediction score which is monotonic with respect to the increasing length of the prefixes of the compared miRNAs and mRNAs (see Figure 10). The score, which is denoted by *min_err*(*u, v*) is computed using dynamic programming.

### The dynamic programming table DP

*for any pair of nodes *(*u*, *v*), *u *∈ *τ*_*M*_, *and v *∈ *τ*_*G*_, *DP *[*u, v*]* holds the target prediction score between the prefix of the (miRNA) sequence spelled by the path from the root of τ*_*M *_*to node u versus the prefix of the (mRNA) sequence spelled by the path from the root of τ*_*G *_*to node v, respectively*.

Let *u' *be the parent of node *u *in *τ*_*M *_and let *α *be the character labeling the edge between them. Similarly, let *v' *be the parent of *v *in *τ*_*G *_and let *β *be the character labeling the edge between them. If we traverse the trees in pre-order traversal, then (*u'*, *v'*), (*u'*, *v*), and (*u, v'*) are computed before (*u, v*).

The target prediction score of (*u*, *v*) is computed using the recurrence formula given below:

where *miRNA_gap *and *mRNA_gap *are penalties for inserting a gap on the miRNA side of the duplex or on the mRNA side of the duplex, respectively, and *match*(*α*, *β*) is the dis-complementarity penalty between the characters *α *and *β *(equal to 0 if *α *and *β *are complementary, and a positive score otherwise).

Note that mismatches and gaps increase *min_err*. Using the restrictions on miRNA target detection based on sequence complementarity, we can bound *min_err *with a threshold *k*. Thus, an mRNA sequence *T *has a chance of being a target of miRNA sequence *P *if *min_err *of *P *and *T *is at most *k*.

### Observation

*The score min_err*(*u, v*) *is monotone with respect to an increase in the prefix length of P and T *(see *k *and *k' *in Figure 10).

Several features that are related to the hybridization pattern between the miRNA and its targets are incorporated into this algorithm. There is an option (a) to require a seed of a given length and position (perfect complementarity region) in the hybridization between the miRNA and the mRNA, (b) to restrict the number of *GU *pairs that are formed in the duplex, and (c) to restrict the size of each bulge formed in the duplex. The thresholds that we use for these features in our work are derived from the literature describing experimentally verified miRNA targets (see the Results Section).

The computation of *min_err*(*u, v*) is done in pre-order traversal of the trees. Pruning is performed while traversing the mRNA trie. The calculation is stopped at each node *v *of *τ*_*G *_where *min_err*(*u, v*) >*k*, or one of the above listed restrictions is violated. When the algorithm reaches a leaf *u *in *τ*_*M*_, it updates *V *(*u*) according to the mRNA sequences that are located in the non-pruned nodes of *τ*_*G*_. At this point a hybridization structure between the miRNA represented by leaf *u *and the corresponding mRNA target sequences is calculated using the *RNAduplex *program [39]. Duplexes with a normalized free energy score (i.e., the duplex energy normalized by the energy score of the miRNA bound to its perfect complement) less than a given user threshold are filtered. After performing the DP, we are left with an miRNA tree where every leaf node *v *is associated with the set of mRNAs which are its predicted targets, as maintained in the *T*(*v*) vector.

We note that the filter described in this section is stand-alone, and can be used independently on other species with different parameters.

#### Filter efficiency measurements

We demonstrate the efficiency of applying our filter on the dataset, described in the Datasets subsection, with three sets of parameters as detailed in Table 4. Each UTR was partitioned into sliding windows of size 30 nts, with a sliding offset of 1 nts between frames, resulting in 7356844 subsequences. Since the *RNAduplex *procedure is by far the most time consuming component of our method, we measured the efficiency of our filter in terms of the number of calls to *RNAduplex*.

**Table 4:** The efficiency of the filter on three different sets of parameters.

Constraints						(*A*) *RNAduplex *calls in the naive method	(*B*) *RNAduplex *calls by our method	(*C*)% of saved calls
			
Seed location	No. of GU pairs		Maximal bulge size					
					
	in seed	total	on miRNA	on mRNA	Maximal no. of errors			
2-8	1	4	6	6	6	1819306	1108226	39%

2-8	1	2	6	6	3	1819306	140920	92%

2-7	0	4	6	6	6	480099	267087	45%

We define by the *naive method *a method that calls *RNAduplex *when there is a seed complementarity, under the constraints of seed location and number of GU pairs in the seed (see columns 1 and 2 in Table 4). Columns (A) and (B) contain the number of *RNAduplex *calls for the naive method and for our method, respectively, and column (C) contains the percent of calls saved by our method.

In all three data sets there is a great reduction in the number of *RNAduplex *calls. The middle row demonstrates a much bigger save in number of calls by our method. This is mainly because of the strong constraint applied on the number of allowed errors (column 6). The reduction in the number of calls to *RNAduplex *is due to the compression of the sequences of both miRNAs and mRNAs into prefix trees and the strong pruning that we apply during the calculation of the hybridization score, accompanied with the constraints that we apply on potential duplexes.

### Hypergeometric p-value

For a given module obtained for a pair <*A, C*>, the hypergeometric p-value is computed as follows: Let *n *be the total number of genes in the dataset and *c *the number of genes that belong to the GO category *C *(denoted as *N *and *C *in Figure 3, respectively). Let *t *be the number of mutual targets of the miRNAs in the module and *tc *the number of genes among them that also belong to *C *(the latter is the result of the enumeration algorithm). The *hypergeometric p-value *of this module is:

### Pseudocode of the enumeration algorithm

   **input **: *Genes *- list of genes from a certain GO category. Every gene *x *in this list has a set of human and viral miRNAs targeting it, denoted as *x.h_miRs *and *x.v_miRs*, respectively.

   **input **: *h_miRs*, *v_miRs *- full list of human and viral miRNAs.

   **output**: Enumeration tree

   root← new Node();

   dummy← new Node();

   dummy.*h_miRs← h_miRs*;

   dummy.*v_miRs← v miRs*;

   root.children.add(dummy);

   **foreach ***gene *∈* Genes ***do**

      node← appendSiblings(*gene*);

      root.children.add(*node*);

   **end**

**Algorithm 1**: construct tree

   **input **: gene - a new gene to be inserted into the tree

   **input **: root - the root node of the enumeration tree

   **input **: *q*_1_, *q*_2 _- quorum restriction on human and viral miRNAs

   **output**: Creates a new node and appends to it (as its children) all its siblings

   node = new Node(*gene*);

   **foreach ***child ∈ root:children ***do**

      sibling← copy(child);

      **foreach ***leaf ∈ sibling:leaves ***do**

         size1← leaf.*h_miRs.size*;

         leaf.*h_miRs← *Intersect(*leaf.h_miRs, gene.h_miRs*);

         size2← leaf.*v_miRs.size*;

         leaf.*v_miRs← *Intersect(*leaf.v_miRs; gene.v_miRs*);

         **if ***size(leaf.h_miRs) == size1 and size(leaf.v_miRs) == size2 ***then**

            /* Delete due to the containment      */

            Delete the path from the leaf in child subtree;

         **end**

         **if ***leaf.h_miRs.size < q1 or leaf.v_miRs.size < q2 ***then**

            /* Prune due to the quorum restrictions      */

            Delete the path from the leaf in sibling subtree;

         **end**

      **end**

      node.children.add(*sibling*);

   **end**

   return node;

**Algorithm 2**: appendSiblings
